# Log-Linear Model Based Behavior Selection Method for Artificial Fish Swarm Algorithm

**DOI:** 10.1155/2015/685404

**Published:** 2015-01-26

**Authors:** Zhehuang Huang, Yidong Chen

**Affiliations:** ^1^School of Mathematics Sciences, Huaqiao University, Quanzhou 362021, China; ^2^Cognitive Science Department, Xiamen University, Xiamen 361005, China

## Abstract

Artificial fish swarm algorithm (AFSA) is a population based optimization technique inspired by social behavior of fishes. In past several years, AFSA has been successfully applied in many research and application areas. The behavior of fishes has a crucial impact on the performance of AFSA, such as global exploration ability and convergence speed. How to construct and select behaviors of fishes are an important task. To solve these problems, an improved artificial fish swarm algorithm based on log-linear model is proposed and implemented in this paper. There are three main works. Firstly, we proposed a new behavior selection algorithm based on log-linear model which can enhance decision making ability of behavior selection. Secondly, adaptive movement behavior based on adaptive weight is presented, which can dynamically adjust according to the diversity of fishes. Finally, some new behaviors are defined and introduced into artificial fish swarm algorithm at the first time to improve global optimization capability. The experiments on high dimensional function optimization showed that the improved algorithm has more powerful global exploration ability and reasonable convergence speed compared with the standard artificial fish swarm algorithm.

## 1. Introduction

Artificial fish swarm algorithm [1, 2] is a new evolutionary algorithm proposed in 2002 to imitate the fish behaviors such as praying, swarming, and following. For many optimization problems, artificial fish swarm algorithm can produce high-quality solution within a reasonable computation time and relatively stable convergence characteristics.

Compared with traditional evolutionary algorithm, artificial fish swarm algorithm has some attractive characteristics such as simple in principle, good robustness, and tolerance of parameter setting [3]. In recent years, a series of papers have focused on the application of AFSA, such as resource leveling [4], robot task allocation [5, 6], neural network [7], image segmentation [8], fault diagnosis in mine hoist [9], image reconstruction [10], data clustering [11–13], PID controller parameters [14], and other areas [15–21].

However, AFSA algorithm also showed some unsatisfactory aspects in practical applications, such as premature convergence and poor ability in global optimization. At the same time, the experiences of group members are not used for the next moves. To solve the problem, some improved algorithms [22–25] have been proposed. Although the methods mentioned above can improve the performance to some extent, they still cannot get satisfactory results for some applications.

At the selection of behaviors, we signed by the log-linear model of thinking. In statistics, linear model is used in different ways according to the context. Log-linear model [26] is a mathematical model that takes the form of a function whose logarithm is a polynomial function of the parameters of the model.

In this paper, we proposed a behavior selection algorithm based on log-linear model. The advantages of log-linear model are the ability to easily blend multiple features. Using the log-linear model can enhance decision making ability of behavior selection.

This paper is organized as follows. In Section 2, related work is presented. The background of artificial fish swarm algorithm is introduced in Section 3. The improved algorithm based on log-linear model and some new behaviors of fishes are presented in Section 4. In Section 5, some experimental tests, results, and conclusions are given.  Section 6 concludes the paper.

## 2. Related Work

In recent years, researchers have made some attempts to improve the performance of artificial fish swarm algorithm. In [22], chaos optimization is first employed to initialize the position of individual artificial fish and applied to obtain the neighborhood of the global optimum solution. Gao et al. reported an improved artificial fish swarm algorithm with crossover operator based on parents' characteristics [23]. In [24], the concept of ecological niche is also being introduced to overcome the shortcoming of traditional artificial fish swarm algorithm to obtain optimal solution. Rocha et al. reported an augmented Lagrangian methodology with a stochastic population based algorithm, which solves a sequence of simple bound global optimization subproblems using a fish swarm intelligent algorithm [25].

Some researchers have tried to improve the performance of AFSA by combining different algorithm with AFSA. An improved AFSO (IAFSO) [27] is proposed to train forward neural network using a hybrid of artificial fish swarm algorithm and particle swarm optimization. In [28], some new ideas are proposed which focus on a set of movements of fishes. Wang and Ma reported a hybrid artificial fish swarm algorithm, which is combined with CF and artificial fish swarm algorithm to solve the Bin packing problem [29]. In [30], quantum rotation gate is used to update the position of artificial fish swarm which employs the quantum nongate to speed up the convergence. The experimental results show that the performance of AFSA is significantly improved. A hybrid algorithm based on particle swarm optimization and artificial fish swarm algorithm [31] is reported which owns a good globally convergent performance with a faster convergent rate. In [32], a simulated annealing artificial fish swarm algorithm is proposed which can obtain better optimization precision and convergence speed.

Log-linear model is a mathematical model which is widely used in part-of-speech tagging, statistical machine translation, and other fields. Och and Ney introduced the log-linear model for statistical machine translation (SMT), in which translation is considered as optimization problem. Its efficiency is comparable to the global method [33]. In [34], the multinomial diversity model (MDM) based on generalized linear models is proposed which can be used for model selection and graphical and numerical interpretation.

In this paper, we proposed an improved algorithm based on log-linear model which can transform the traditional single probability model to adaptive model.

## 3. Introduction to AFSA

**Figure 1:**
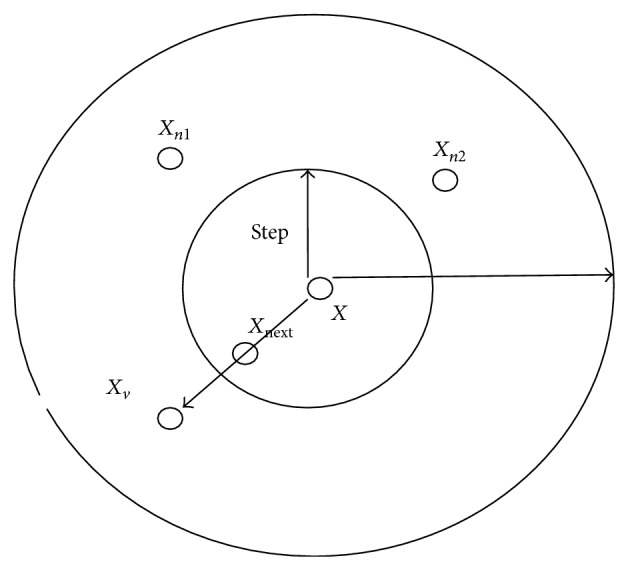
Vision concept of the artificial fish.

Suppose *X* is the state vector of artificial fish swarm. Let *X* = (*x*_1_, *x*_2_,…, *x*_*n*_), where *x*_1_, *x*_2_,…, *x*_*n*_ is status of fishes. The action of artificial fish occurs only in the radius of a circle with vision. The artificial fish realizes external perception by its vision shown in Figure 1.

Suppose *X*_*v*_ is the visual position at some moment; *X*_*v*_ = (*x*_1_^*v*^, *x*_2_^*v*^,…, *x*_*n*_^*v*^). *X*_next_ is the new position. Let Step be the moving step length, Visual represents the visual distance, and *δ* is the crowd factor (0 < *δ* < 1). Then the movement process is represented as

(1) $$\begin{array}{c} X_{v}=X_{i}+\text{Visual}\times \text{rand}\left(\right),\;i\in \left(0,n\right].\\ X_{\text{next}}=X+\frac{X_{v}-X}{\left\|X_{v}-X\right\|}\times \text{Step}\times \text{rand}\left(\right), \end{array}$$

where rand() produces random numbers between 0 and 1.

The basic behaviors of artificial fish are defined as follows.

### 3.1. Prey Behavior

Prey behavior is a basic biological behavior to find food. Suppose *X*_*i*_ is the current state of artificial fish and *X*_*j*_ is a random state in its visual distance, and *Y* is the food concentration:

(2) $$\begin{array}{c} X_{j}=X_{i}+\text{Visual}\times \text{rand}\left(\right). \end{array}$$

If *Y*_*i*_ < *Y*_*j*_, it goes forward a step in this direction:

(3) $$\begin{array}{c} X_{i}^{t+1}=X_{i}^{t}+\frac{X_{j}-X_{i}^{t}}{\left\|X_{j}-X_{i}^{t}\right\|}\times \text{Step}\times \text{rand}\left(\right). \end{array}$$

### 3.2. Swarm Behavior

Suppose *X*_*c*_ is the center position and the number of artificial fish is *n*_*f*_. If *n*_*f*_/*n* < *δ* and *Y*_*c*_ > *Y*_*i*_, it goes forward a step to the companion center; otherwise perform prey behavior:

(4) $$\begin{array}{c} X_{i}^{t+1}=X_{i}^{t}+\frac{X_{c}-X_{i}^{t}}{\left\|X_{c}-X_{i}^{t}\right\|}\times \text{Step}\times \text{rand}\left(\right). \end{array}$$

### 3.3. Follow Behavior

Suppose *X*_max⁡_ is the optimal state from visual neighbors, and the number of partners of *X*_max⁡_ is *n*_*f*_. If *n*_*f*_/*n* < *δ* and *Y*_*j*_ > *Y*_*i*_, it goes forward a step to the companion *X*_*j*_; otherwise perform prey behavior:

(5) $$\begin{array}{c} X_{i}^{t+1}=X_{i}^{t}+\frac{X_{\max}-X_{i}^{t}}{\left\|X_{\max}-X_{i}^{t}\right\|}\times \text{Step}\times \text{rand}\left(\right). \end{array}$$

### 3.4. Move Behavior

Move behavior is a basic behavior to seek food or companions in larger ranges. They can choose a state in the vision and then move towards this state:

(6) $$\begin{array}{c} X_{i}^{t+1}=X_{i}^{t}+\text{Visual}\times \text{rand}\left(\right). \end{array}$$

### 3.5. Leap Behavior

If the fitness function is almost the same or the difference is smaller than a proportion during the given (*m* − *n*) iterations, leap behavior can move to new state to avoid the local extreme values.

If (*Y*_max⁡_^*m*^ − *Y*_max⁡_^*n*^ < *eps*),

(7) $$\begin{array}{c} X_{i}^{t+1}=X_{i}^{t}+\beta \times \text{Visual}\times \text{rand}\left(\right), \end{array}$$

where *eps* is a smaller constant and *β* is a parameter that can make some fishes have other actions.

The artificial fish swarm algorithm mainly includes the following steps.

**Step 1 .**

Initialize the parameters of artificial fish, such as Step, Visual, the number of exploratory, and maximum number of iterations.

**Step 2 .**

Select the optimal value, and recorded in the bulletin.

**Step 3 .**

Perform prey behavior, swarm behavior, follow behavior, move behavior, and leap behavior.

**Step 4 .**

If individual optimum is better than bulletin board, update the optimal value in bulletin board.

**Step 5 .**

If the termination condition is satisfied, output the result; otherwise return to Step 2.

## 4. The Improved Fish Swarm Algorithm Based on Log-Linear Model (LAFSA)

In this section, we proposed a behavior selection algorithm based on log-linear model. Firstly, log-linear model is used to implement a multiprobability adaptive model. A variety of knowledge sources are added to the model in the form of a feature function to enhance decision making ability. Secondly, some new behaviors are proposed to improve the performance of fish swarm algorithm.

### 4.1. Log-Linear Model

Suppose that the state vector of fishes is *X* = (*x*_1_, *x*_2_,…, *x*_*n*_), where *x*_1_, *x*_2_,…, *x*_*n*_ is status of the fishes. The basic form of the behavior selection algorithm based on log-linear model can be defined as

(8) $$\begin{array}{c} P_{r}\left(t\right)=\frac{\sum_{m}^{}\exp\left[\beta_{m}h_{m}\left(t\right)\right]}{\sum_{t}^{}\sum_{m}^{}\exp\left[\beta_{m}h_{m}\left(t\right)\right]}, \end{array}$$

where *h*_*m*_(*t*), (*m* = 1,2,…, *M*) are feature functions and *λ*_*m*_  (*m* = 1,2,…, *M*) are the weight of feature function.

### 4.2. The Selection of Feature Function

The selections of feature functions have a crucial impact on the optimization performance; how to construct feature functions is an important task. In this paper, we choose diversity function, dimensional distribution function, and average distance metrics function as feature function in the experiment.

*(1) Diversity Function*. Diversity function is used to describe the degree of dispersion of the fishes. Diversity function *h*_1_(*t*) describes adaptive diversity of *t*th iteration:

(9) $$\begin{array}{c} h_{1}\left(t\right)=\frac{\sum_{i=1}^{N}\left|f\left(x_{i}\right)-f_{\text{avg}}^{t}\right|}{N*\max_{1\leq j\leq N}\left(\left|f\left(x_{j}\right)-f_{\text{avg}}^{t}\right|\right)}, \end{array}$$

where *f*_avg_^*t*^ means the average fitness value of *t*th iterative and *f* is the fitness value of the current fishes *X*_*i*_. Consider *h*_1_(*t*)∈(0,1]. *h*_1_(*t*) = 1 means the diversity is poor. *h*_1_(*t*) ≪ 1 means the diversity is good.

*(2) Dimensional Distribution Function*. We select dimensional distribution function as feature function:

(10) $$\begin{array}{c} h_{2}\left(t\right)=\frac{\sum_{i=1}^{N}\sqrt{\sum_{d}^{D}{\left(x_{id}-{\overset{-}{x}}_{d}\right)}^{2}}}{N\times \max_{1\leq j\leq N}\left(\sqrt{\sum_{d}^{D}{\left(x_{jd}-{\overset{-}{x}}_{d}\right)}^{2}}\right)}, \end{array}$$

where *N* means the number of particles and ${\overset{-}{x}}_{d}$ means the average value of the *D* dimension component of particles. When *h*_2_(*t*) is less than a threshold, we must employ some strategies to improve the diversity of population.

*(3) Average Distance Metrics Function*. We select average distance metrics function as feature function. Suppose that the distance between the fishes is *d*_*i*,*j*_ = ‖*X*_*i*_ − *X*_*j*_‖, and *i* and *j* are the number of random fishes. Diversity function *h*_3_(*t*) describes average distance of *i*th fishes *X*_*i*_:

(11) $$\begin{array}{c} h_{3}\left(t\right)=\frac{\sum_{j=1}^{N}d_{i,j}}{N\times \max_{1\leq i,j\leq N}\left(d_{i,j}\right)},\;i=1,2,\ldots ,N. \end{array}$$

Additionally, we can choose other functions such as contraction expansion measure as feature function.

### 4.3. New Behaviors

#### 4.3.1. Adaptive Movement Behavior

Suppose that inertia weight vector is *w* = [*w*_1_, *w*_2_,…, *w*_*n*_]; here *w*_*i*_  (*i* = 1,2,…, *n*) is inertia weight of the *i*th dimension, and its value dynamically adjusts according to diversity of fishes. When the diversity of fishes is good, the inertia weight can be set smaller. When the diversity of fishes is good, the inertia weight can be set larger. The inertia weight of *i*th dimension can be set as

(12) $$\begin{array}{c} \omega_{i}=0.6*e^{\left(-\alpha *(1-P_{r}(t))\right)}+0.3,\;i=1,2,\ldots ,n, \end{array}$$

where diversity function *P*_*r*_(*t*) describes adaptive diversity of *t*th iteration. *α* is a constant; we set *α* = 10 in the experiment.

**Figure 2:**
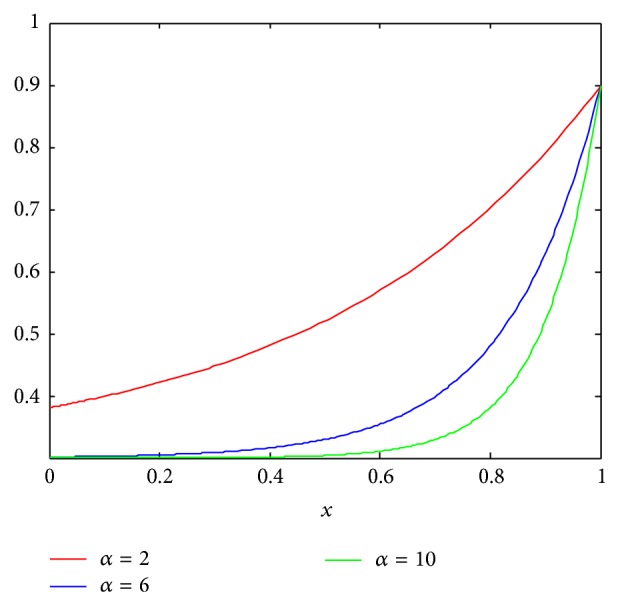
The image of function *f*(*x*) = 0.6∗*e* ^(−*α*∗(1−*x*))^ + 0.3.

The image of function *f*(*x*) = 0.6∗*e* ^(−*α*∗(1−*x*))^ + 0.3 is shown in Figure 2. It can be used to describe the change tendency of parameters *ω*_*i*_.

So the traditional prey behavior, swarm behavior, and follow behavior can be redefined as adaptive movement behavior based on inertia weight. The adaptive movement behavior includes three behaviors: new prey behavior, new swarm behavior, and new follow behavior, which is shown as (13) to (15).

New prey behavior is

(13) $$\begin{array}{c} X_{i}^{t+1}=w_{i}X_{i}^{t}+\left(1-w_{i}\right)\frac{X_{j}-X_{i}^{t}}{\left\|X_{j}-X_{i}^{t}\right\|}\times \text{Step}\times \text{rand}\left(\right). \end{array}$$

New swarm behavior is

(14) $$\begin{array}{c} X_{i}^{t+1}=w_{i}X_{i}^{t}+\left(1-w_{i}\right)\frac{X_{c}-X_{i}^{t}}{\left\|X_{c}-X_{i}^{t}\right\|}\times \text{Step}\times \text{rand}\left(\right). \end{array}$$

New follow behavior is

(15) $$\begin{array}{c} X_{i}^{t+1}=w_{i}X_{i}^{t}+\left(1-w_{i}\right)\frac{X_{\max}-X_{i}^{t}}{\left\|X_{\max}-X_{i}^{t}\right\|}\times \text{Step}\times \text{rand}\left(\right). \end{array}$$

If (*P*_*r*_(*t*) > *R*_1_), perform adaptive movement behavior; else perform traditional prey behavior, swarm behavior, and follow behavior, where *R*_1_ is diversity threshold value, and we set *R*_1_ = 0.6 in the experiment.

#### 4.3.2. Population Inhibition Behavior

In artificial fish swarm algorithm, the convergence speed of later stages is too slow; a lot of fishes perform invalid search which wastes much time. So we introduce population inhibition behavior to accelerate the convergence speed.

After a period of evolution, the big fishes will eat small fishes, and the occupied space of small fishes will be cleared.

**Figure 3:**
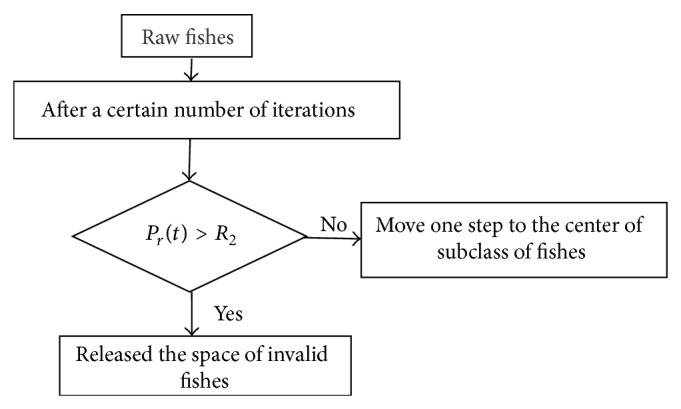
The population inhibition behavior of artificial fish swarm.

The population inhibition behavior of artificial fish swarm is shown in Figure 3, where *R*_2_ is diversity threshold value, and we set *R*_2_ = 0.85 in the experiment.

#### 4.3.3. Population Expansion Behavior

Population inhibition behavior can improve the convergence speed, but it may lead to poor global exploration ability. So we introduce population expansion behavior to maintain a certain population size.

**Figure 4:**
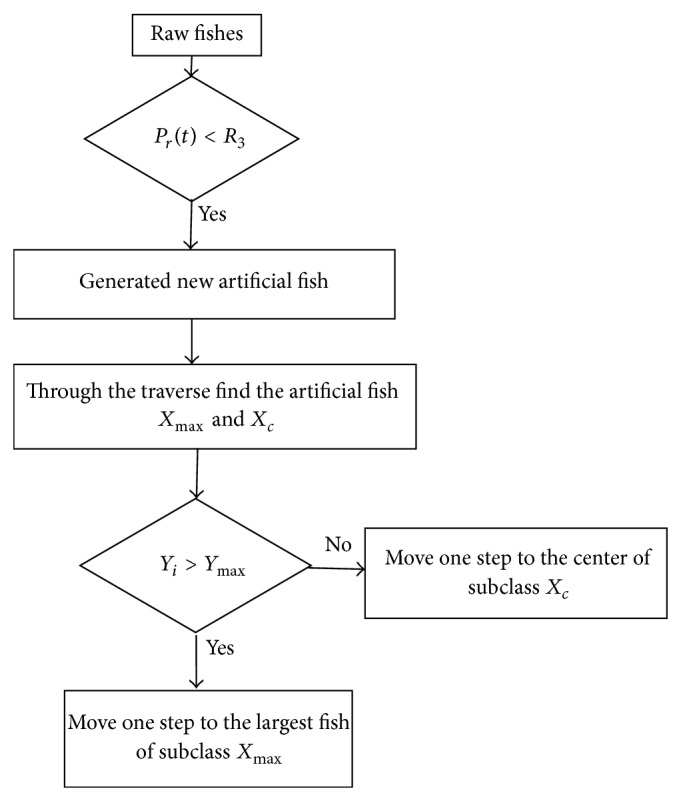
The population expansion behavior of artificial fish swarm.

For fish *i*, it can generate subswarm which is in line with the normal distribution. The generation of new fishes can be set as

(16) $$\begin{array}{c} X_{i}^{\text{new}}=X_{i}+\text{random}\left(-l*\text{Step},l*\text{Step}\right), \end{array}$$

where *l* is an integer constant. The population expansion behavior of artificial fish swarm is shown in Figure 4, where *R*_3_ is diversity threshold value, and we set *R*_3_ = 0.2 in the experiment.

For the new artificial fishes, firstly find *X*_max⁡_ with the largest objective function value, and if *Y*_*i*_ < *Y*_max⁡_, then move one step to the center of subclass *X*_*c*_; otherwise move one step to the largest fish of subclass *X*_max⁡_.

### 4.4. The Behavior Selection Algorithm Based on Log-Linear Model

The improved algorithm is shown in Algorithm 1.

**Figure 5:**
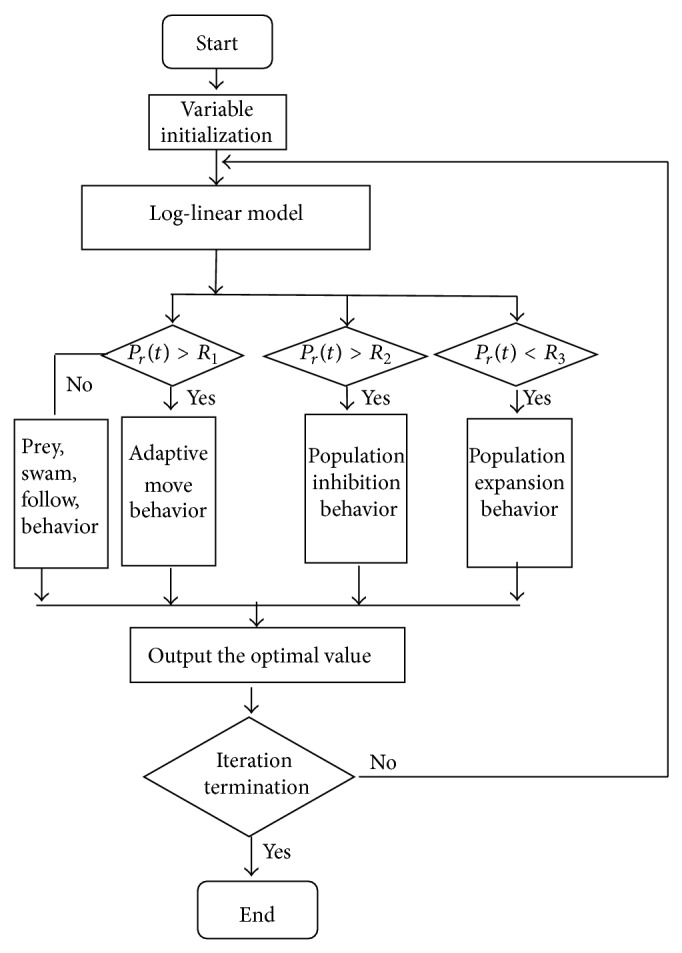
Log-linear model based artificial fish swarm algorithm.

The overall structure of the improved algorithm is shown in Figure 5.

## 5. Experiment

A set of unconstrained benchmark functions was used to investigate the effect of the improved algorithm which is shown in Table 1.

**Figure 6:**
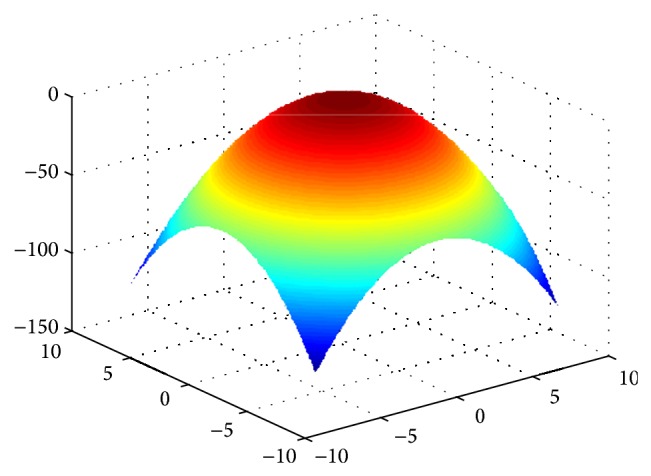
The image of Sphere function.

Sphere function is a single-peak function, we can find the optimal value to be 0 through the analysis for function expression, and the function image is shown in Figure 6.

**Figure 7:**
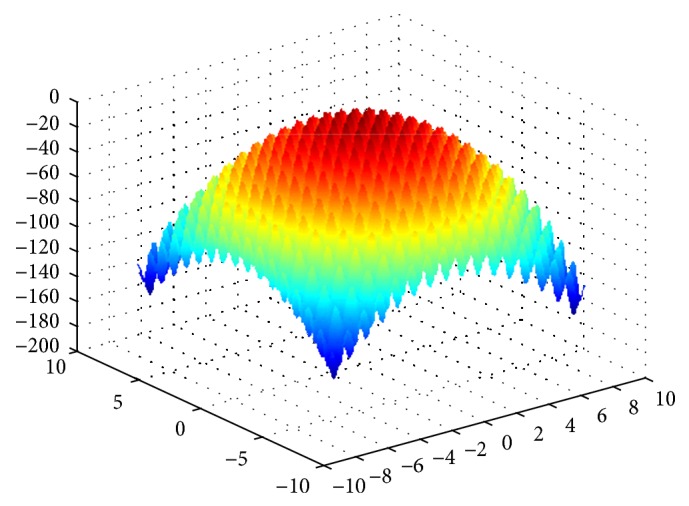
The image of Rastrigin function.

Rastrigin function is a multipeak function, we can find the optimal value to be 0 through the analysis for function expression, and the function image is shown in Figure 7.

**Figure 8:**
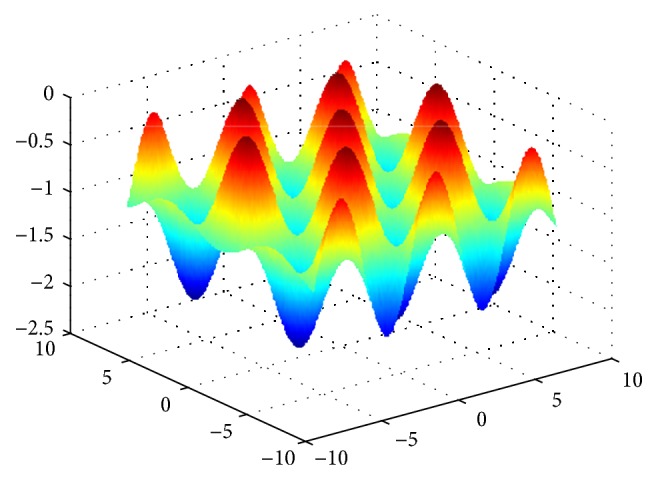
The image of Griewank function.

Griewank function is a multipeak function, we can find the optimal value to be 0 through the analysis for function expression, and the function image is shown in Figure 8.

**Figure 9:**
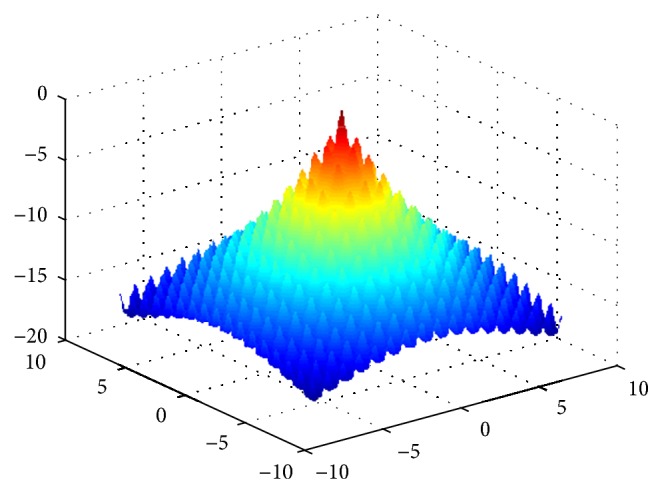
The image of Ackley function.

Ackley function is a multipeak function, we can find the optimal value to be 0 through the analysis for function expression, and the function image is shown in Figure 9.

**Figure 10:**
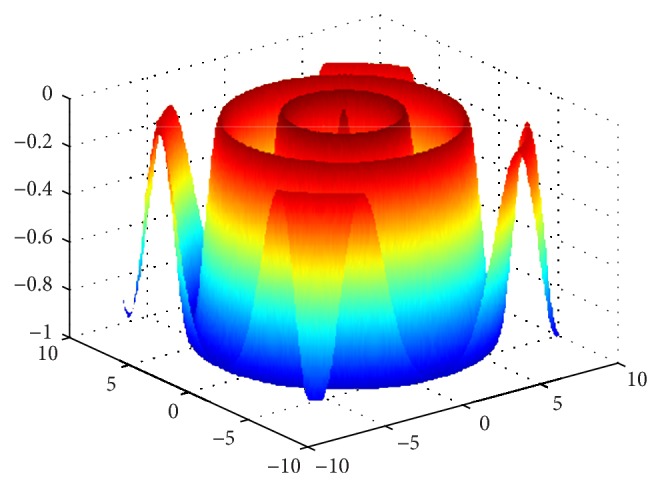
The image of Shaffer function.

Shaffer function is a multipeak function, we can find the optimal value to be 0 through the analysis for function expression, and the function image is shown in Figure 10.

The parameter setting of test algorithm is shown in Table 2.

For comparison, we use five strategies:

1. AFSA: basic artificial fish swarm algorithm,
2. AFSA + AMB: artificial fish swarm algorithm with adaptive movement behavior based on log-linear model,
3. AFSA + PIB: artificial fish swarm algorithm with population inhibition behavior based on log-linear model,
4. AFSA + PEB: artificial fish swarm algorithm with population expansion behavior based on log-linear model,
5. LAFSA: improved artificial fish swarm algorithm with hybrid behavior (adaptive movement behavior, population inhibition behavior, and population expansion behavior) based on log-linear model.

We used mean (average optimal value), times (execution time), SD (standard deviation), and iterations (average convergence iterations number) as evaluation indicators.

The comparison of different methods is shown in Table 3 to Table 5. Each point is made from average values of over 30 repetitions. The dimension of Sphere function, Rastrigin function, Griewank function, and Ackley function is taken as 10 dimensions. Shaffer function is taken as 2 dimensions.

We can see from Table 3, for Sphere function, Rastrigin function, and Ackey function, that we can improve the optimal value compared with the standard AFSA. For Griewank function and Shaffer function, the optimal value of the two algorithms is the same.

So the adaptive inertia weight can effectively reflect the diversity of fish swarm. Adaptive movement behavior with reasonable setting according to diversity of fish swarm can improve the performance.

From Table 4, we can find out that the convergence time of AFSA + PIB is greatly reduced as expected for all the five functions. The number of fishes is gradually reduced which can avoid useless search, so the time can be reduced. But we can see that optimal value is slightly inferior to the standard algorithm.

For all the five functions, the accuracy obtained in AFSA + PEB is significantly improved because the introduction of population expansion behavior can improve global optimization capability. But at the same time, the execution time inevitably increases compared with the standard AFSA algorithm.

So different behavior has both advantages and disadvantages, from an overall point of view, and we must select different behaviors according to different condition.

From Table 5, for Sphere function, Rastrigin function, Griewank function, and Ackley function, LAFSA algorithm can effectively improve the accuracy such that the optimal value obtained is much closer to the theoretical one and the accuracy is improved compared with the standard AFSA algorithm. On the convergence speed and algorithm execution time, there is a significant improvement as expected for all the five functions.

So the new behaviors of fish presented in this paper are essential for better performance. At the same time, the behavior selection based on log-linear model can enhance decision making ability of behavior selection.

**Figure 11:**
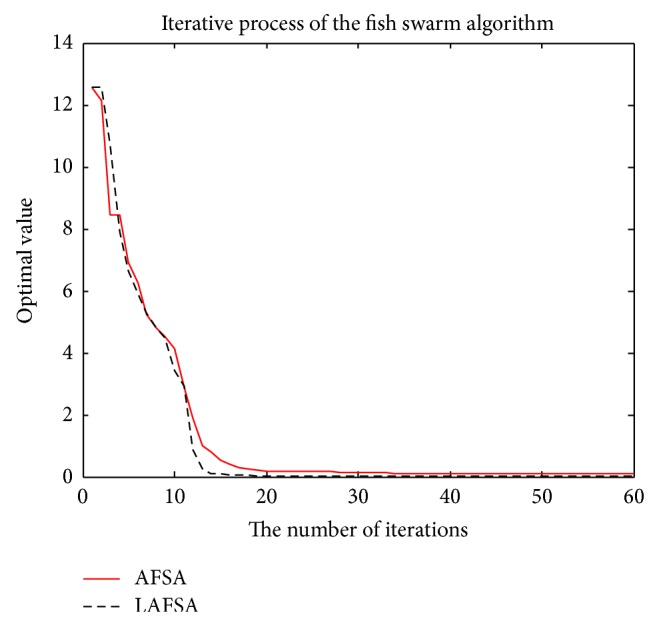
Sphere function.

**Figure 12:**
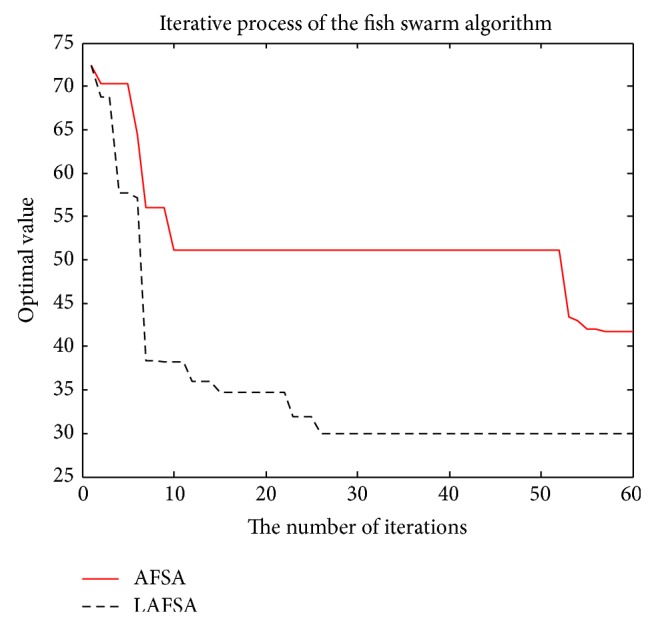
Rastrigin function.

**Figure 13:**
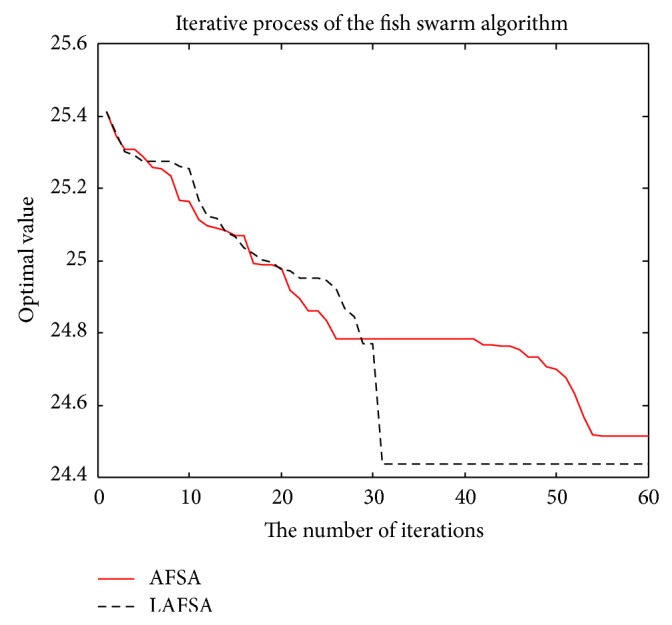
Griewank function.

**Figure 14:**
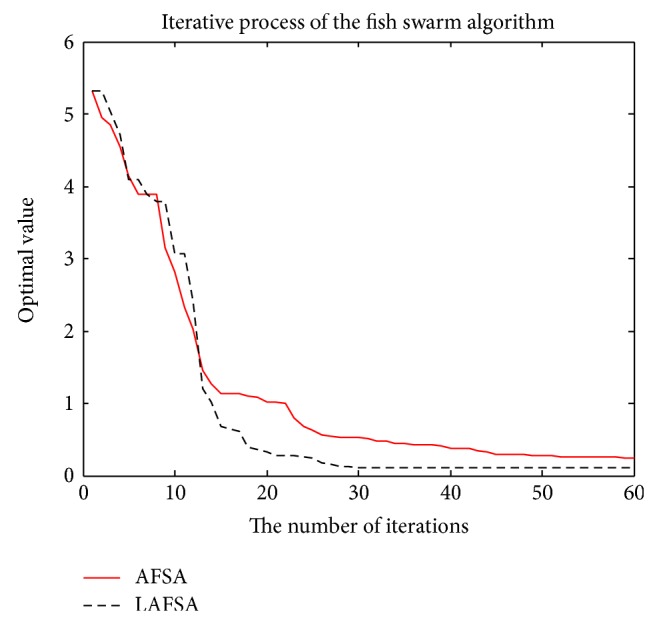
Ackley function.

**Figure 15:**
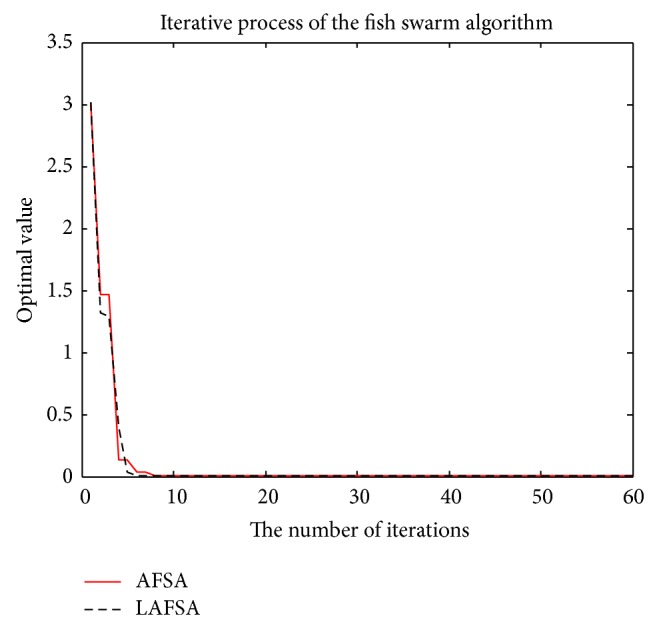
Shaffer function.

The comparison of the two methods with convergent curves is shown in Figures 11, 12, 13, 14, and 15. Comparing with AFSA, the LAFSA algorithm has both global search ability and fast convergence speed.

## 6. Conclusions

In this paper, we present an improved artificial fish swarm algorithm based on log-linear model. This is the first effort to introduce log-linear model and some new behavior to artificial fish swarm algorithm. Experiments show that the improved algorithm has more powerful global exploration ability with reasonable convergence speed.

We got the following conclusions.

1. Using the log-linear model, the traditional single probability model can be transformed into multiprobability adaptive model, which can efficiently use the advantages of a variety of probabilistic models and enhance decision making ability of behavior selection.
2. Adaptive movement behavior with reasonable setting according to diversity of fish swarm can improve the performance of basic artificial fish swarm algorithm.
3. Population inhibition behavior is introduced which can greatly accelerate the convergence speed.
4. Population expansion behavior can improve global optimization capability and avoid premature convergence.

From the experiment, we can find out that the behavior of fishes is essential for better performance. We will introduce some new behaviors in future work and apply this idea to other optimization tasks.

## Figures and Tables

**Algorithm 1:**
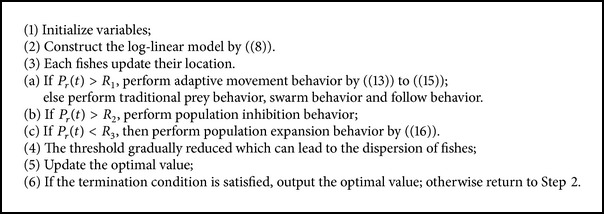
Improved AFSA based on log-linear model.

**Table 1:** Functions used to test the effects of improved algorithm.

Function	Function expression	Optimal value
Sphere function	$f_{1}\left(x\right)=\overset{n}{\underset{t=1}{\sum }}x_{t}^{2}$	0

Rastrigin function	$f_{2}\left(x\right)=\overset{n}{\underset{t=1}{\sum }}\left(x_{t}^{2}-10\cos\left(2\pi x_{t}\right)+10\right)$	0

Griewank function	$f_{3}(x)=\frac{1}{4000}\overset{n}{\underset{t=1}{\sum }}{\left(x_{t}-100\right)}^{2}-\prod_{t=1}^{n}\cos\left(\frac{x_{t}-100}{\sqrt{t}}\right)+1$	0

Ackley function	$f_{4}(x)=20+e-20e^{-0.2\sqrt{\sum_{t=1}^{n}{x_{t}}^{2}/n}}-e^{\sum_{t=1}^{n}\cos\;\left(2\pi x_{t}\right)/n}$	0

Shaffer's function	$f_{5}\left(x\right)=0.5+\frac{{\left(\sin\sqrt{{x_{1}}^{2}+{x_{2}}^{2}}\right)}^{2}-0.5}{{\left(1+0.001\left({x_{1}}^{2}+{x_{2}}^{2}\right)\right)}^{2}}$	0

**Table 2:** The parameter setting of test algorithm.

Algorithm	Fish number	Visual	Delta	Step	Number of iterations
AFSA	100	2.85	9	1	60
LAFSA	100	2.85	9	1	60

**Table 3:** The performances of AFSA and AFSA + AMB.

Algorithm	AFSA		AFSA + AMB	
	Mean	Time (s)	Mean	Time (s)
Sphere	0.017	3.02	0.016	3.13
Rastrigin	42.7	3.52	34.6	3.43
Griewank	24.56	2.21	24.56	2.56
Ackley	0.25	3.93	0.14	3.18
Shaffer	0.0097	1.79	0.0097	1.72

**Table 4:** The testing performance comparison among of AFSA, AFSA + PIB, and AFSA + PEB.

Algorithm	AFSA		AFSA + PIB		AFSA + PEB	
	Mean	Time (s)	Mean	Time (s)	Mean	Time (s)
Sphere	0.017	3.02	0.036	1.49	0.013	4.59
Rastrigin	42.7	3.52	45.3	1.55	22.3	4.58
Griewank	24.56	2.21	26.25	1.33	18.63	3.51
Ackley	0.25	3.93	0.33	1.96	0.16	5.19
Shaffer	0.0097	1.79	0.051	1.38	0	3.22

**Table 5:** The performances of AFSA and LAFSA.

Algorithm	AFSA				LAFSA			
	Mean	SD	Time (s)	Iterations	Mean	SD	Time (s)	Iterations
Sphere	0.017	0.0048	3.02	21	0.014	0.0023	1.88	15
Rastrigin	42.7	7.54	3.52	55	29.6	5.31	2.23	28
Griewank	24.56	4.91	2.21	57	24.45	3.04	1.35	32
Ackley	0.25	0.072	3.93	45	0.18	0.054	2.456	31
Shaffer	0.0097	0.00013	1.79	8	0.0097	0.00011	1.51	8

## References

[1] Li X. L., Feng S. H., Qian J. X. (2004). Parameter tuning method of robust pID controller based on artificial fish school algorithm. *Chinese Journal of Information and Control*.

[2] Li X. L., Lu F., Tian G. H., Qian J. X. (2004). Applications of artificial fish school algorithm in combinatorial optimization problems. *Chinese Journal of Shandong University (Engineering Science)*.

[3] Li X. L., Qian J. X. (2003). Studies on artificial fish swarm optimization algorithm based on decomposition and coordination techniques. *Chinese Journal of Circuits and Systems*.

[4] Tian W. J., Tian Y. An improved artificial fish swarm algorithm for resource leveling.

[5] Zheng T. X., Li J. Q. Multi-robot task allocation and scheduling based on fish swarm algorithm.

[6] Tian W. J., Liu J. C. An improved artificial fish swarm algorithm for multi robot task scheduling.

[7] Zhang M. F., Shao C., Li F. C., Gan Y., Sun J. M. Evolving neural network classifiers and feature subset using artificial fish swarm.

[8] Tian W. J., Geng Y., Liu J. C., Ai L. Optimal parameter algorithm for image segmentation.

[9] Wang C.-J., Xia S.-X. (2010). Application of probabilistic causal-effect model based artificial fish-swarm algorithm for fault diagnosis in mine hoist. *Journal of Software*.

[10] Chen D. Y., Shao L., Zhang Z., Yu X. Y. An image reconstruction algorithm based on artificial fish-swarm for electrical capacitance tomography system.

[11] Cheng Y. M., Jiang M. Y., Yuan D. F. Novel clustering algorithms based on improved artificial fish swarm algorithm.

[12] Chu X. L., Zhu Y., Shi J. T., Song J. Q. Method of image segmentation based on fuzzy C-means clustering algorithm and artificial fish swarm algorithm.

[13] Li X. A clustering algorithm based on artificial fish school.

[14] Luo Y., Wei W., Wang S. X. Optimization of PID controller parameters based on an improved artificial fish swarm algorithm.

[15] Yazdani D., Nabizadeh H., Kosari E. M., Toosi A. N. (2011). Color quantization using modified artificial fish swarm algorithm. *AI 2011: Advances in Artificial Intelligence: Proceedings of the 24th Australasian Joint Conference, Perth, Australia, December 5–8, 2011*.

[16] Huang Z. H., Chen Y. D. (2014). A two-stage exon recognition model based on synergetic neural network. *Computational and Mathematical Methods in Medicine*.

[17] Bing D., Wen D. Scheduling arrival aircrafts on multi-runway based on an improved artificial fish swarm algorithm.

[18] Qi A. L., Ma H. W., Liu T. A weak signal detection method based on artificial fish swarm optimized matching pursuit.

[19] Zhang J., Shen L. (2014). An improved fuzzy *c*-means clustering algorithm based on shadowed sets and PSO. *Computational Intelligence and Neuroscience*.

[20] Song X., Wang C. R., Wang J., Zhang B. A hierarchical routing protocol based on AFSO algorithm for WSN.

[21] Liu T., Qi A. L., Hou Y. B., Chang X. T. Feature optimization based on artificial fish-swarm algorithm in intrusion detections.

[22] Guo W., Fang G. H., Huang X. F. An improved chaotic artificial fish swarm algorithm and its application in optimizing cascade hydropower stations.

[23] Gao X. Z., Wu Y., Zenger K., Huang X. A knowledge-based Artificial Fish-swarm Algorithm.

[24] Ma X. Application of adaptive hybrid sequences niche artificial fish swarm algorithm in vehicle routing problem.

[25] Rocha A. M. A., Martins T. F. M. C., Fernandes E. M. G. P. (2011). An augmented Lagrangian fish swarm based method for global optimization. *Journal of Computational and Applied Mathematics*.

[26] Och F. J. Minimum error rate training in statistical machine translation.

[27] Wang C. R., Zhou C. L., Ma J. W. An improved artificial fish swarm algorithm and its application in feed-forward neural networks.

[28] Fernandes E. M. G. P., Martins T. F. M. C., Rocha A. M. A. C. Fish swarm intelligent algorithm for bound constrained global optimization.

[29] Wang L., Ma L. J. A hybrid artificial fish swarm algorithm for Bin-packing problem.

[30] Zhu K. C., Jiang M. Y. Quantum artificial fish swarm algorithm.

[31] Xiu-Xi W., Hai-Wen Z., Yong-Quan Z. Hybrid artificial fish school algorithm for solving ill-conditioned linear systems of equations.

[32] Jiang M. Y., Cheng Y. M. Simulated annealing artificial fish swarm algorithm.

[33] Och F. J., Ney H. (2004). The alignment template approach to statistical machine translation. *Computational Linguistics*.

[34] De'ath G. (2012). The multinomial diversity model: linking Shannon diversity to multiple predictors. *Ecology*.

